# Gender Differences in 3-Month Outcomes of Erenumab Treatment—Study on Efficacy and Safety of Treatment With Erenumab in Men

**DOI:** 10.3389/fneur.2021.774341

**Published:** 2021-12-16

**Authors:** Raffaele Ornello, Carlo Baraldi, Simona Guerzoni, Giorgio Lambru, Matteo Fuccaro, Bianca Raffaelli, Astrid Gendolla, Piero Barbanti, Cinzia Aurilia, Sabina Cevoli, Valentina Favoni, Fabrizio Vernieri, Claudia Altamura, Antonio Russo, Marcello Silvestro, Elisabetta Dalla Valle, Andrea Mancioli, Angelo Ranieri, Gennaro Alfieri, Nina Latysheva, Elena Filatova, Jamie Talbot, Shuli Cheng, Dagny Holle, Armin Scheffler, Tomáš Nežádal, Dana Čtrnáctá, Jitka Šípková, Zuzana Matoušová, Lucia Sette, Alfonsina Casalena, Maurizio Maddestra, Stefano Viola, Giannapia Affaitati, Maria Adele Giamberardino, Francesca Pistoia, Uwe Reuter, Simona Sacco

**Affiliations:** ^1^Neuroscience Section, Department of Applied Clinical Sciences and Biotechnology, University of L'Aquila, L'Aquila, Italy; ^2^Department of Biomedical, Metabolic and Neural Sciences, School in Neurosciences, University of Modena and Reggio Emilia, Modena, Italy; ^3^Medical Toxicology - Headache and Drug Abuse Research Center, Department of Biomedical, Metabolic and Neural Sciences, University of Modena and Reggio Emilia, Modena, Italy; ^4^The Headache Service, Guy's and St Thomas' National Health Service (NHS) Foundation Trust, London, United Kingdom; ^5^Department of Neurology, Charité - Universitätsmedizin Berlin, Berlin, Germany; ^6^Praxis Gendolla, Essen, Germany; ^7^Headache and Pain Unit, Istituto di Ricovero e Cura a Carattere Scientifico (IRCCS) San Raffaele, Rome, Italy; ^8^San Raffaele University, Rome, Italy; ^9^Istituto di Ricovero e Cura a Carattere Scientifico (IRCCS) Istituto delle Scienze Neurologiche di Bologna, Bologna, Italy; ^10^Headache and Neurosonology Unit, Policlinico Universitario Campus Bio-Medico, Rome, Italy; ^11^Headache Center, Department of Medical, Surgical, Neurological, Metabolic, and Aging Sciences, University of Campania “Luigi Vanvitelli”, Naples, Italy; ^12^Headache Centre, Ospedale S. Antonio Abate, ASST Valle Olona, Gallarate, Italy; ^13^Headache Centre, Division of Neurology and Stroke Unit, “A. Cardarelli” Hospital, Naples, Italy; ^14^Sechenov First Moscow State Medical University (Sechenov University), Moscow, Russia; ^15^Southwest Neurology Audit and Research Group, Department of Neurology, Derriford Hospital, Plymouth, United Kingdom; ^16^Department of Neurology, Alfred Health, Melbourne, VIC, Australia; ^17^Department of Neurology, West German Headache Center, University Hospital Essen, Essen, Germany; ^18^Department of Neurology, Military University Hospital Prague, First Faculty of Medicine Charles University, Prague, Czechia; ^19^Department of Neurology, Motol University Hospital Prague, First Faculty of Medicine Charles University, Prague, Czechia; ^20^Department of Neurology, “G. Mazzini” Hospital, Teramo, Italy; ^21^Department of Neurology, “F. Renzetti” Hospital, Chieti, Italy; ^22^Department of Neurology, “S. Pio da Pietrelcina” Hospital, Chieti, Italy; ^23^Headache Center, Geriatrics Clinic, Department of Medicine and Science of Aging and Center for Advanced Studies and Technology, G. D'Annunzio University, Chieti, Italy; ^24^Universitätsmedizin Greifswald, Greifswald, Germany

**Keywords:** migraine, erenumab, gender, migraine treatment, men, real-world evidence

## Abstract

**Objective:** We reported gender-specific data on the efficacy and safety of erenumab, a monoclonal antibody antagonizing the calcitonin gene-related peptide (CGRP) receptor.

**Methods:** Our pooled patient-level analysis of real-world data included patients treated with erenumab and followed up for 12 weeks. We considered the following outcomes at weeks 9–12 of treatment compared with baseline: 0–29%, 30–49%, 50–75%, and ≥75% responder rates, according to the decrease in monthly headache days (MHDs), rate of treatment stopping, change in MHDs, monthly migraine days (MMDs), monthly days of acute medication and triptan use, and Headache Impact Test-6 (HIT-6) score from baseline to weeks 9–12. Outcomes were compared between men and women by the chi-squared test or *t*-test, as appropriate. An analysis of covariance (ANCOVA) was performed to identify factors influencing the efficacy outcomes.

**Results:** We included 1,410 patients from 16 centers, of which 256 (18.2%) were men. Men were older than women and had a lower number of MHDs at baseline. At weeks 9–12, compared with baseline, 46 (18.0%) men had a ≥75% response, 75 (29.3%) had a 50–74% response, 35 (13.7%) had a 30–49% response, and 86 (33.6%) had a 0–29% response, while 14 (5.5%) stopped the treatment. The corresponding numbers for women were 220 (19.1%), 314 (27.2%), 139 (12.0%), 402 (34.8%), and 79 (6.8%). No gender difference was found in any of the outcomes. The ANCOVA showed that gender did not influence the efficacy of outcomes.

**Conclusion:** We found that erenumab is equally safe and effective in men compared with women after 12 weeks.

## Introduction

Migraine is a recurrent headache disorder that can be considered a gender disease, as it affects more women than men. In detail, the prevalence of migraine is 2-3-folds higher in women than in men from adolescence to 50 years of age (1). Several factors, including the provoking action of estrogens, genetic heritability, and psychosocial factors such as pain catastrophizing, are thought to determine the higher prevalence and burden of migraine in women when compared with men (2). Sex hormones and genetic factors determine a brain dimorphism in which the female brain is more susceptible to migraine when compared with the male brain (3). For the above reasons, the proportion of men with migraine is much lower than that of women. Men account for <20% of patients included in observational and interventional studies on migraine (2). This proportion is too low to allow adequate generalizability of overall results to the male gender.

Monoclonal antibodies acting on the calcitonin gene-related peptide (CGRP) pathway are the first approved preventive drugs targeting a migraine-specific mechanism (4–6). The efficacy and safety of monoclonal antibodies acting on the CGRP pathway have been largely proven by several randomized controlled trials (7–10) and real-world studies (11–22). However, the proportion of men included in those studies did not allow the performance of subgroup analyses according to gender. Assessing the gender-specific response to treatments targeting the CGRP is an interesting research question, given the sexual dimorphism of the trigeminovascular system that is responsible for CGRP release (23).

With the present study, we aimed to provide reliable gender-specific results on the efficacy and safety of erenumab, a monoclonal antibody acting on the CGRP receptor. To achieve this aim, we collected a large dataset of real-world data.

## Materials and Methods

### Inclusion Criteria and Study Population

A study on efficacy and safety of treatment with erenumab in men (ESTEEMen) was a pooled patient-level analysis of real-world data referring to treatment with erenumab. To be considered for the study, all centers had to meet the following criteria:

- Having performed real-life studies on erenumab treatment for migraine prevention, approved by the local Ethics Committees, for which patients already signed an informed consent if required by the local regulation.
- Using migraine diaries to collect patients' data.
- Being able to share a patient-level database of patients treated with erenumab reporting efficacy and safety endpoints.
- Minimal follow-up duration of 12 weeks.

Real-life studies that did not report outcome variables, including migraine/headache days and acute medication use, were not considered for the present study. Claims data and patient surveys were also excluded from the present analysis.

A literature search containing the terms “erenumab” and “real-life” or “real-world” was performed in PubMed in January 2021. From that search, we selected eligible studies and contacted the corresponding authors to ask for participation in the ESTEEMen study. The authors also referred to personal contacts to search for eligible centers.

The pooled analysis of the ESTEEMen study was approved by the Internal Review Board of the University of L'Aquila with protocol number 07/2021, and local ethical approval to pool data was obtained if needed.

### Variables and Outcomes

All included patients were followed up for 12 weeks, irrespective of treatment discontinuation. Baseline was considered as the 4 weeks preceding the start of erenumab treatment, while outcomes were assessed at weeks 9–12 of treatment and compared with the baseline. Weeks 9–12 were chosen because data were available from all centers and because that timepoint was common to most randomized controlled trials of erenumab (7–9).

Monthly headache days (MHDs), monthly migraine days (MMDs), and monthly days of use of acute medication and triptans were collected in all centers by using headache diaries. Mean Headache Impact Test-6 (HIT-6) scores were also compared between baseline and weeks 9–12. We classified the categories of response as 0–29%, 30–49%, 50–74%, and ≥75%, according to the reduction in MHDs at weeks 9–12 when compared with baseline. We also reported the proportion of patients stopping the treatment because of inadequate response, adverse events, or loss to follow-up. Stopping of treatment due to inadequate response occurred based on the preference of patients, as erenumab treatment was meant to be continued for at least 12 weeks.

### Statistical Analysis

Descriptive statistics were reported as numbers and proportions and means and SDs or SEs, as appropriate. We performed a chi-square test with adjustment by linear trend data to compare categorical variables, and a Student *t*-test to compare continuous variables according to gender. All analyses used the intent-to-treat population, which included all patients treated with at least one dose of erenumab.

We performed univariate comparisons of baseline variables between men and women, using the chi-square test or Student *t*-test, as appropriate. Baseline variables were considered for the analyses if available for at least two-thirds of patients; no imputation was done for missing baseline data.

Efficacy outcomes included the proportion of 0–29%, 30–49%, 50–74%, and ≥75% responders and mean change in continuous variables (MHDs, MMDs, days of use of acute medication and triptans, and HIT-6 scores) from baseline to weeks 9–12. Safety outcomes included the proportion of patients stopping the treatment within the first 12 weeks, with reasons, and the proportion of subjects reporting adverse events. Given the absence of gender-specific studies on erenumab, all outcomes were considered as exploratory and no adjustment for multiple comparisons was performed. To impute missing diary data of patients stopping the treatment, we repeated the last available observation according to a “last observation carried forward” approach.

To assess the influence of baseline patients' characteristics, including gender, on the efficacy of erenumab, and to adjust for possible differences between men and women, we performed an analysis of covariance (ANCOVA) for each of the continuous efficacy variables, considering as covariates the variables with *P* < 0.1 at the univariate comparison between men and women. A propensity score matching between men and women was initially considered and then abandoned due to persistent imbalance despite matching.

No sample size calculation was performed, as we used a convenience sample based on the available data. Two-tailed *P* for significance was set at <0.05. Statistical analyses were conducted using SPSS version 20.

## Results

Sixteen of 18 invited centers agreed to provide data. The number of patients included by each center is reported in Supplementary Table 1. A total of 1,410 patients (256 males; 18.2%) were included.

The univariate comparison between men and women showed that, at baseline, men were older and had lesser MHDs when compared with women; men also had a lower prevalence of chronic migraine and a lower impact of migraine on everyday activities, as shown by the lower HIT-6 scores (Table 1). Acute medication consumption was comparable in men and women (Table 1).

**Table 1:** Baseline characteristics and gender comparisons in the present study.

	**All (*n* = 1,410)**	**Men (*n* = 256)**	**Women (*n* = 1,154)**	***P-*value**
Age, mean ± SD	48.3 ± 11.5	49.6 ± 1.1	48.0 ± 11.3	**0.038**
Duration of migraine history (years), mean ± SD	24.7 ± 14.6	23.9 ± 23.6	24.8 ± 13.0	0.769
Chronic migraine, *n* (%)	1,036 (73.5)	169 (66.0)	867 (75.1)	**0.002**
Medication overuse, *n* (%)	733 (52.0)	133 (52.0)	600 (52.0)	0.830
Failed prior preventive drugs, mean ± SD	5.5 ± 3.0	5.5 ± 3.0	5.5 ± 2.9	0.941
Monthly headache days, mean ± SD	21.2 ± 7.5	20.2 ± 7.4	21.4 ± 7.5	**0.028**
Monthly migraine days, mean ± SD	17.1 ± 8.0	16.8 ± 7.7	17.2 ± 8.0	0.453
Monthly acute medication days, mean ± SD	16.2 ± 8.5	16.1 ± 8.4	16.2 ± 8.5	0.862
Monthly triptan use days, mean ± SD	10.9 ± 9.8	11.2 ± 9.9	10.9 ± 9.8	0.768
HIT-6 score, mean ± SD	66.7 ± 6.6	65.0 ± 6.1	67.1 ± 6.7	**<0.001**

*P values in bold highlight significant results. HIT-6, Headache Impact Test-6; SD, standard deviation*.

**Figure 1:**
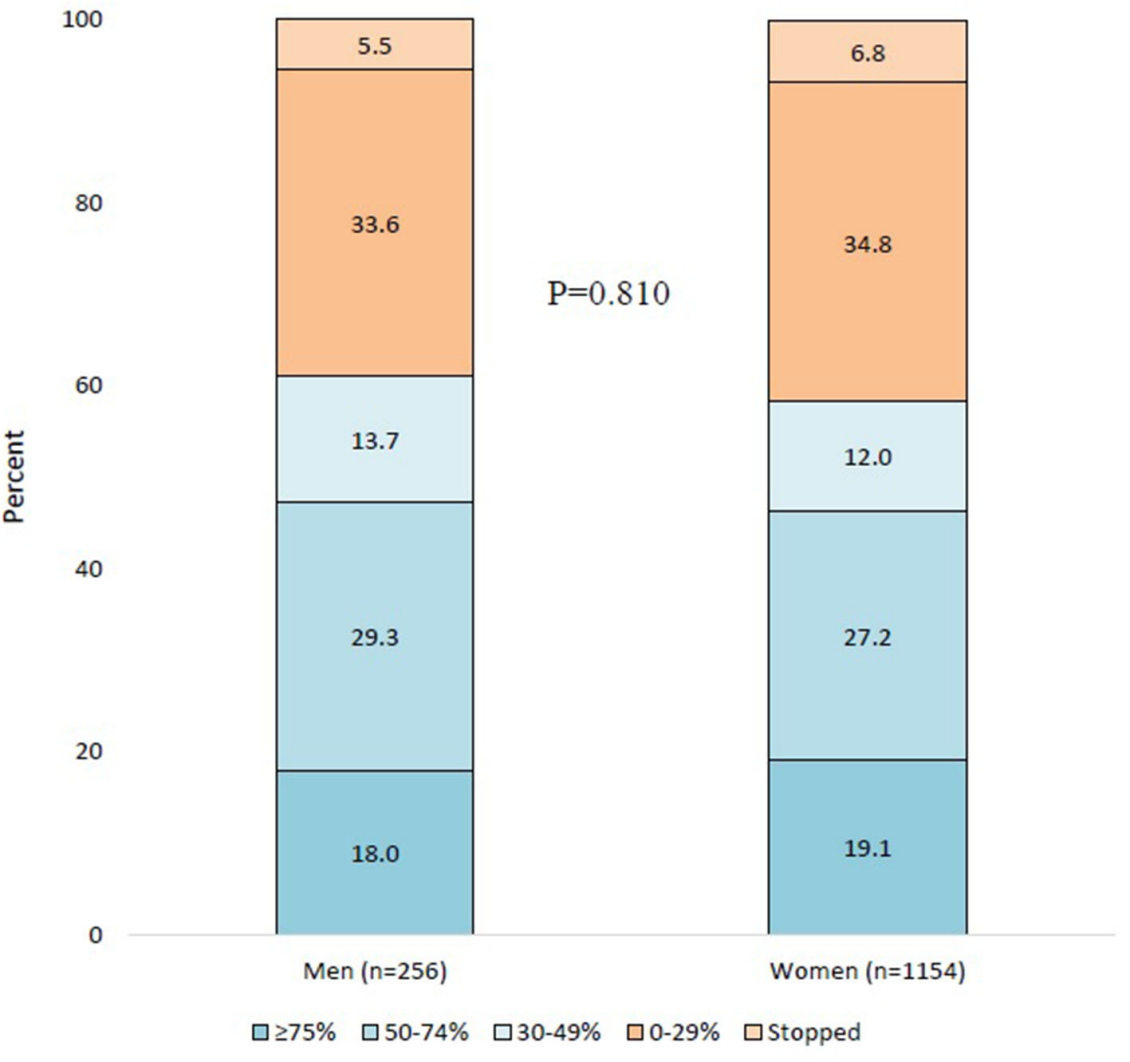
Categories of decrease in monthly migraine days at weeks 9–12 compared with baseline in men and women. The *P*-value refers to the gender comparison in category distribution.

At weeks 9–12, compared with baseline, 46 (18.0%) men had a ≥75% response, 75 (29.3%) had a 50–74% response, 35 (13.7%) had a 30–49% response, and 86 (33.6%) had a 0–29% response, while 14 (5.5%) had stopped the treatment. The corresponding numbers for women were 220 (19.1%), 314 (27.2%), 139 (12.0%), 402 (34.8%), and 79 (6.8%). The response and stopping rates were comparable between men and women (*P* = 0.810; Figure 1).

**Figure 2:**
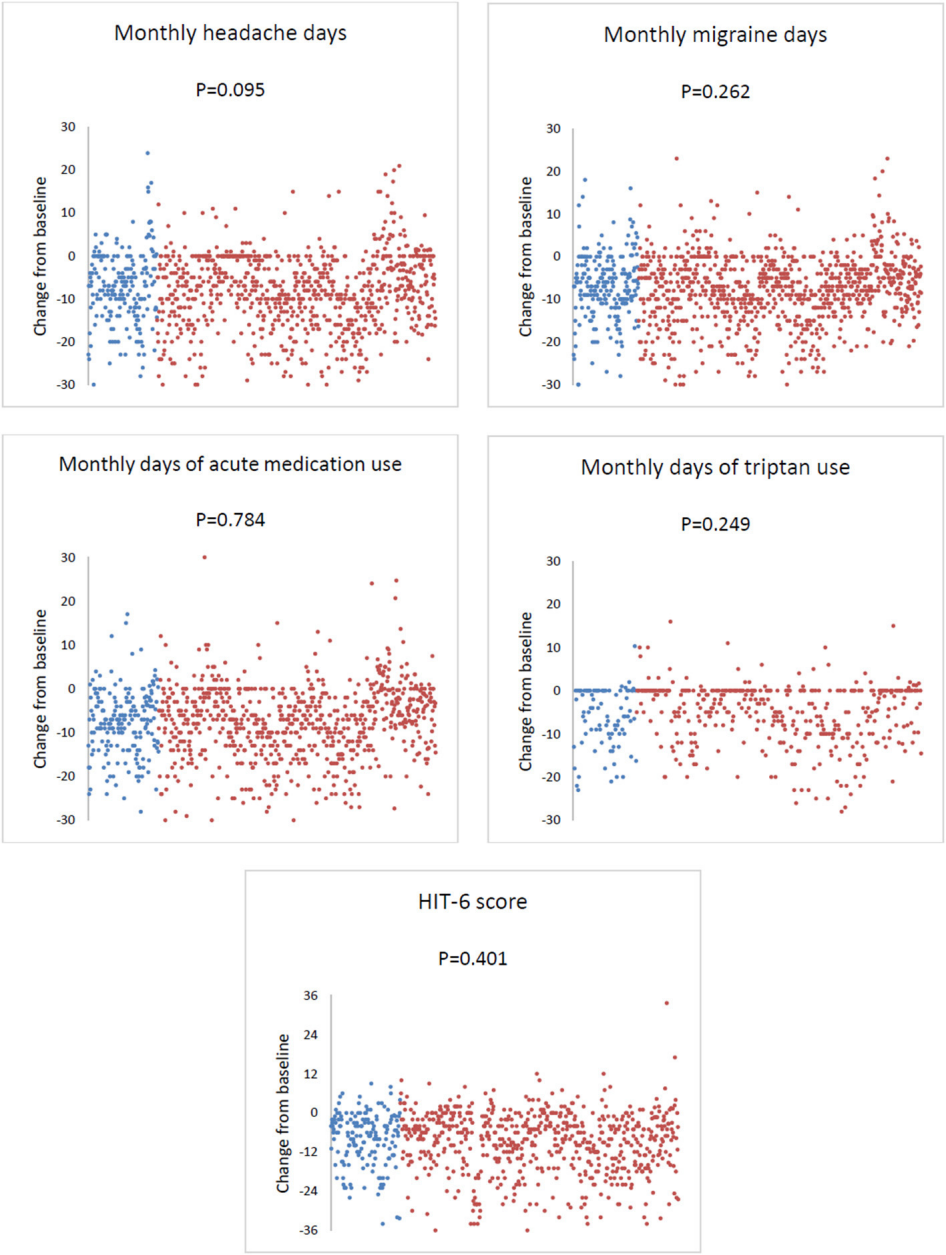
Change in monthly headache days, monthly migraine days, acute medication use, and Headache Impact Test-6 (HIT-6) score from baseline to weeks 9–12 of erenumab use in men and women. Each dot represents a patient. Blue dots indicate men, while red dots indicate women.

At weeks 9–12, compared with baseline, the mean ± SE change in MHDs was −6.9 ± 0.6 in men and −7.9 ± 0.3 in women (*P* = 0.095); the change in MMDs was −7.1 ± 0.5 in men and −7.7 ± 0.3 in women (*P* = 0.262); the change in monthly days of acute medication was −7.6 ± 0.5 in men and −7.5 ± 0.3 in women (*P* = 0.784), while that of monthly days of triptan use was −6.0 ± 0.7 in men and −5.1 ± 0.3 in women (*P* = 0.249); HIT-6 score change was −8.4 ± 0.7 in men and −9.1 ± 0.4 in women (*P* = 0.401; Figure 2).

Table 2 reports details about the ANCOVA. The analyses showed that the change in MHDs was influenced by age (*F* = 7.852; *P* = 0.005) and baseline MMDs (*F* = 15.164; *P* < 0.001); change in MMDs was influenced by baseline MHDs (*F* = 11.633; *P* = 0.001) and baseline MMDs (*F* = 147.827; *P* < 0.001); change in days of acute medication was influenced by baseline MMDs (*F* = 27.624; *P* < 0.001) and baseline HIT-6 score (*F* = 7.107; *P* = 0.008); change in days of triptan use was influenced by age (*F* = 7.295; *P* = 0.007), baseline MHDs (*F* = 6.982; *P* = 0.009), and MMDs (*F* = 34.207; *P* < 0.001); and change in HIT-6 score was influenced by baseline MHDs (*F* = 38.074; *P* < 0.001), baseline MMDs (*F* = 8.874; *P* = 0.003), and baseline HIT-6 (*F* = 127.031; *P* < 0.001). Notably, gender did not influence any of the outcomes.

**Table 2:** Analysis of covariance for factors interacting with response to erenumab.

	***F***	***B***	**95% CI**	***P-*value**
**Change in monthly headache days**				
Intercept	1.047	3.199	−3.533 to 9.932	0.351
Gender (men vs. women)	0.527	0.514	−0.876 to 1.904	0.468
Age	7.852	−0.072	−0.122 to −0.021	**0.005**
Baseline monthly headache days	2.385	−0.089	−0.201 to 0.024	0.123
Baseline monthly migraine days	15.164	−0.201	−0.303 to −0.100	**<0.001**
Baseline HIT-6 score	0.766	−0.041	−0.134 to 0.051	0.382
**Change in monthly migraine days**				
Intercept	0.211	1.096	−4.968 to 7.160	0.723
Gender (men vs. women)	0.908	0.607	−0.644 to 1.859	0.341
Age	0.070	−0.042	−0.087 to 0.003	0.066
Baseline monthly headache days	11.633	0.176	0.075 to 0.277	**0.001**
Baseline monthly migraine days	147.827	−0.565	−0.657 to −0.474	**<0.001**
Baseline HIT-6 score	0.070	−0.011	−0.094 to 0.072	0.791
**Change in days of acute medication**				
Intercept	4.064	6.806	0.036 to 13.576	**0.049**
Gender (men vs. women)	0.010	0.073	−1.330 to 1.476	0.919
Age	2.556	−0.042	−0.093 to 0.010	0.110
Baseline monthly headache days	0.093	0.018	−0.096 to 0.131	0.761
Baseline monthly migraine days	27.624	−0.277	−0.380 to −0.173	**<0.001**
Baseline HIT-6 score	7.107	−0.126	−0.219 to −0.033	**0.008**
**Change in days of triptan use**				
Intercept	0.701	−2.781	−10.562 to 5.000	0.483
Gender (men vs. women)	1.111	−0.966	−2.767 to 0.836	0.292
Age	7.295	−0.079	−0.137 to −0.022	**0.007**
Baseline monthly headache days	6.982	0.171	0.044 to 0.297	**0.009**
Baseline monthly migraine days	34.207	−0.326	−0.436 to −0.217	**<0.001**
Baseline HIT-6 score	0.884	0.054	−0.059 to 0.166	0.348
**Change in HIT-6 score**				
Intercept	47.864	26.405	18.862 to 33.948	**<0.001**
Gender (men vs. women)	0.293	−0.436	−2.016 to 1.145	0.588
Age	0.515	−0.021	−0.077 to 0.036	0.473
Baseline monthly headache days	38.074	0.403	0.275 to 0.531	**<0.001**
Baseline monthly migraine days	8.874	−0.174	−0.289 to −0.059	**0.003**
Baseline HIT-6 score	127.031	−0.593	−0.697 to −0.490	**<0.001**

*P values in bold highlight significant results*.

At the end of the 12-week observation period, mean ± SD of MHDs (9.6 ± 7.5 vs. 9.5 ± 7.9; *P* = 0.825), MMDs (13.2 ± 9.0 vs. 13.7 ± 9.5; *P* = 0.467), days of acute medication (8.9 ± 6.8 vs. 9.4 ± 7.6; *P* = 0.375), days of triptan use (5.4 ± 5.2 vs. 6.2 ± 7.3; *P* = 0.233), and HIT-6 score (56.6 ± 9.1 vs. 58.1 ± 9.5; *P* = 0.060) did not differ between men and women; 30 men (11.7%) and 175 women (15.2%) had an adverse event, and the most frequent adverse event was constipation in both genders (7.0% in men and 8.9% in women; Supplementary Table 2). Five patients, all women, stopped erenumab treatment due to adverse events.

## Discussion

Reporting gender differences in diseases is important in modern medicine to plan an adequately targeted management. However, in the field of migraine, reporting gender differences in the clinical presentation and response to treatments is difficult due to the far higher prevalence of migraine in women than in men. In the present study, we created a large international collaboration to pool a significant number of men treated with erenumab. The efficacy and safety of erenumab were comparable between men and women.

We found a slightly higher burden of migraine at baseline in women than in men, characterized by a higher prevalence of chronic migraine, a higher number of MHDs at baseline, and higher headache-related disability. However, most characteristics of migraine, and especially MMDs and days of acute medication at baseline, were comparable in men and women. Besides, the high number of patients could have led to significant differences that were likely not relevant on clinical grounds. Population-based evidence shows that migraine is more severe and more disabling in women than in men in the overall population of migraineurs (24). However, patients treated with erenumab represent a selection from that population. In real-world practice, erenumab treatment is prescribed to patients with the most severe forms of migraine, regardless of gender. Hence, we expected to find that men and women had many similar characteristics in our study. According to our findings, erenumab was equally effective in men and women. The absolute proportion of patients with adverse events was slightly higher in women than in men; however, the difference was minimal and non-significant. Notably, we did not find any gender-specific safety concerns.

The comparable efficacy of erenumab in men and women contrasts with the sexual dimorphism of the trigeminovascular system, which is responsible for the release of CGRP and thus the generation of migraine pain. Animal studies proved that the trigeminal ganglion, which is central to the trigeminovascular system, has receptors for female sex hormones (23, 25); besides, the exogenous administration of estrogen compounds can potentiate CGRP release (26). A positive correlation between the levels of estrogen and those of CGRP was also demonstrated in a human study (27). Given the suggestion of a gender-specific CGRP expression, a different response to CGRP blockade in men when compared with women was expected but was not found. Notably, previous studies investigating CGRP release from the trigeminovascular system in animal models did not consider the possible effect of monoclonal antibodies. Those drugs may act with mechanisms that are independent of the release of sex hormones, therefore being equally effective in men and women. However, our study was not designed to prove any biological hypothesis, as it was not interventional, and we did not measure any level of female sex hormones or CGRP in the study population. The measurement of functional biomarkers of migraine in humans is an emerging trend (28) and could provide useful insights when assessing the effect of anti-migraine drugs.

The multicenter design of the present study ensures generalizability to the overall population of patients treated with erenumab in real-world practice. Our data are also the first gender-specific analysis on patients with migraine treated with a monoclonal antibody acting on the CGRP pathway. Our results can be useful in clinical practice to discuss with patients the possible therapeutic options and their anticipated results. However, our study also has limitations. The retrospective design of the study only allowed the inclusion of a limited set of variables; thus, we could not control for all the potential differences between men and women. The observational nature of the present study could also not exclude potential biases. Besides, the observation period was rather short (12 weeks), and gender differences in the response to erenumab might have emerged over a longer time period.

## Conclusions

We showed that erenumab, a monoclonal antibody acting on the CGRP receptor, was equally safe and effective in men when compared with women after 12 weeks. We reported the first gender-specific data on a preventive drug that was designed for migraine. A gap of knowledge should be filled regarding the gender differences in response to migraine treatments and their clinical implications.

## Data Availability Statement

Requests to access the anonymized datasets should be directed to Simona Sacco, simona.sacco@univaq.it; Raffaele Ornello, raffaele.ornello@univaq.it.

## Ethics Statement

Pooling of previously collected data for the present collaborative study was approved by the Internal Review Board of the University of L'Aquila with protocol number 07/2021. All centers had their former data collections approved by the local ethics committees if necessary, according to the local regulations; written informed consent was obtained from patients.

## Author Contributions

RO and SS contributed to the conception and design of the study. RO and LS organized the database. RO and CB performed the statistical analysis and wrote the first draft of the manuscript. SG, GL, MF, BR, AG, PB, CAu, CAl, SCe, VF, FV, ARu, MS, ED, AM, ARa, GAl, NL, EF, JT, SCh, DH, AS, TN, DČ, JŠ, ZM, LS, AC, MM, SV, GAf, MG, FP, UR, and SS performed a critical review of the manuscript. All the authors contributed to manuscript revision, read, and approved the submitted version.

## Funding

This study received funding from Novartis Farma SpA. The funder was not involved in the study design, collection, analysis, interpretation of data, the writing of this article, or the decision to submit it for publication.

## Conflict of Interest

RO reported personal fees from Novartis, Teva, and Eli Lilly, and had non-financial relationships with Allergan/AbbVie, Novartis, and Teva. SS reported personal fees and non-financial support from Allergan, Abbott, Eli Lilly, Novartis, and Teva; personal fees from Medscape; and others from Bayer, Pfizer, Medtronic, Starmed, Bristol–Myers Squibb, and Daiichi Sankyo. BR reported research grants from Novartis, and personal fees from Allergan, Lilly, Novartis, and Teva. UR received honoraria for consulting and lectures from Amgen, Allergan, Abbvie, Eli Lilly, Lundbeck, Novartis, electroCore, Medscape, StreaMedUp, and Teva, and research funding from the German Federal Ministry of Education and Research and Novartis. GAf reported competing interests with Novartis. MG reported competing interests with Helsinn Healthcare and Uriach Italy. GL received speaker honoraria, funding for travel, and honoraria for participation in advisory boards sponsored by Allergan, TEVA, Lilly, and Novartis; he also received speaker honoraria and funding for travel from electroCore, Nevro Corp, and Autonomic Technologies. NL reported personal fees from Novartis and Allergan. EF reported personal fees from Novartis and Teva. CB and SG received honoraria from Allergan, Teva, Eli Lilly, and Novartis. DH reported personal fees from Novartis, Teva, Eli Lilly, Allergan, Hormosan, Zuellig Pharma, and Bayer. ARa received speaker honoraria from Novartis, Teva, and Eli Lilly. SCe received travel grants, honoraria for advisory boards, speaker panels, or clinical investigation studies from Novartis, Teva, Lilly, Allergan, Ibsa, Amgen, and Lundbeck. VF received honoraria as a speaker or for participating in advisory boards from Eli Lilly, Novartis, and Teva. ARu received speaker honoraria from Allergan, Lilly, Novartis, and Teva. MS received speaker honoraria from Lilly, Novartis, and Teva. FV received travel grants, honoraria for advisory boards, speaker panels, or clinical investigation studies from Allergan, Eli-Lilly, Novartis, and Teva. PB received travel grants, honoraria for advisory boards, speaker panels, or clinical investigation studies from Alder, Allergan, Bayer, ElectroCore, Eli Lilly, GSK, Lusofarmaco, MSD, Novartis, Stx-Med, Teva, and Visufarma. CAu received travel grants from Eli Lilly, FB-Health, Lusofarmaco, and Teva. The remaining authors declare that the research was conducted in the absence of any commercial or financial relationships that could be construed as a potential conflict of interest.

## Publisher's Note

All claims expressed in this article are solely those of the authors and do not necessarily represent those of their affiliated organizations, or those of the publisher, the editors and the reviewers. Any product that may be evaluated in this article, or claim that may be made by its manufacturer, is not guaranteed or endorsed by the publisher.
