# Supplementary material for: Gender Differences in 3-Month Outcomes of Erenumab Treatment—Study on Efficacy and Safety of Treatment With Erenumab in Men
Source: Front Neurol. 2021 Dec 16;12:774341. doi: 10.3389/fneur.2021.774341 (PMC8717149; doi:10.3389/fneur.2021.774341)
Supplement: Supplementary file 1 [file Table_1.DOCX]

Supplementary Material

**Supplementary Table 1**. Contribution of each center.

| **Center** | **Overall (n, %)** | **Men (n, %*)** |
| --- | --- | --- |
| Essen (West Germany) | 159 (11.3) | 28 (17.6) |
| Modena | 148 (10.5) | 28 (18.9) |
| London (St Thomas’) | 139 (9.9) | 25 (18.0) |
| Abruzzo*** | 138 (9.8) | 17 (12.3) |
| Rome (San Raffaele) | 128 (9.1) | 30 (23.4) |
| Prague** | 127 (9.0) | 11 (8.7) |
| Plymouth | 98 (6.9) | 17 (17.3) |
| Bologna | 92 (6.5) | 29 (31.5) |
| Essen (Praxis Gendolla) | 71 (5.0) | 15 (21.1) |
| Berlin | 68 (4.8) | 8 (11.8) |
| Melbourne | 65 (4.6) | 8 (12.3) |
| Naples (Vanvitelli) | 61 (4.3) | 14 (23.0) |
| Gallarate | 39 (2.8) | 10 (25.6) |
| Naples (Cardarelli) | 31 (2.2) | 4 (12.9) |
| Rome (Campus Biomedico) | 28 (2.0) | 9 (32.1) |
| Moscow (Sechenov) | 18 (1.3) | 3 (16.7) |
|  |  |  |
| Total | 1410 (100.0) | 256 (18.2) |

*proportion over the total cases of each center

**including Military and Motol Hospitals

***including the centers of L’Aquila, Avezzano, Teramo, Chieti, Lanciano, and Vasto

**Supplementary Table 2**. Adverse event categories in men and women after 12 weeks of erenumab treatment.

| **Variable** | **All (n=1410)** | **Men (n=256)** | **Women (n=1154)** | **P value** |
| --- | --- | --- | --- | --- |
| Constipation, n (%) | 121 (8.6) | 18 (7.0) | 103 (8.9) | 0.328 |
| Pain at injection site, n (%) | 5 (0.4) | 0 | 5 (0.4) | 0.291 |
| Flu-like symptoms, n (%) | 12 (0.9) | 1 (0.4) | 11 (1.0) | 0.375 |
| Other, n (%) | 80 (5.7) | 11 (4.3) | 69 (6.0) | 0.293 |
|  |  |  |  |  |
| Any adverse event, n (%) | 205 (14.5) | 30 (11.7) | 175 (15.2) | 0.157 |
| Adverse events causing treatment stopping, n (%) | 5 (0.4) | 0 | 5 (0.4) | 0.291 |
